# Developing a Practical Medical Educator Training Program: Meeting A Need in Faculty Development

**DOI:** 10.15694/mep.2020.000271.1

**Published:** 2020-12-04

**Authors:** Isabel Dominguez, Ann Zumwalt

**Affiliations:** 1 Boston University School of Medicine

**Keywords:** Educator training program, teacher training program, faculty development, mentoring, program development, interprofessional collaboration, career advancement

## Abstract

This article was migrated. The article was marked as recommended.

Faculty who teach in medical schools typically do so because of their knowledge and expertise in their field, yet few receive training in best practices in teaching. Educator development programs that help faculty enhance their teaching skills while continuing to fulfill their existing professional responsibilities can help address this gap. Such programs may be developed and implemented locally by individuals within the institution. This guide is intended for individuals who are interested in developing educator training programs but who lack experience in program development. The article describes practical strategies for designing, implementing, and evaluating a collaborative program to teach skills and best practices in medical education. Key themes in program design, program implementation, and program evaluation and dissemination include appropriate goal setting, setting clear expectations, strong communication, and the benefits of diversity in collaboration. Educator training programs provide enhanced teaching skills and opportunities for career advancement for participants at all career stages, which in turn benefits the institution and the medical profession.

## Introduction

Basic science and clinician faculty in healthcare education are typically asked to teach because of their knowledge and expertise in their field, yet few have received training in best practices for how to teach that material ( Ciaccia, 2011; Peluso and Hafler, 2011; Chen *et al.*, 2017). While many healthcare educators succeed without formal training as teachers, there are numerous scenarios in which formalized teacher training would benefit existing educators.

Educator training programs are especially useful for healthcare educators being asked to teach in new contexts. For example, individuals who have active teaching roles may be asked to respond to external pressures such as curriculum changes that require modification to their teaching style. Two examples in modern medical curricula that are increasingly common are the integration of the basic sciences with clinical sciences, and the growing emphasis on interactivity in instructional design. Both of these trends require educators of all types to evolve and grow in how they teach their area of expertise. In addition, individuals may take on or be placed in new roles that require their teaching skills to evolve, such as working with a different team of educators, directing a new course, or working under new leadership. Educators may also be motivated to improve their teaching, either in response to student evaluations or for the sake of personal excellence. In addition to active medical educators, these training programs are useful for early career trainees who are interested in becoming educators and can benefit from mentoring in how best to succeed in the educational setting of the institution ( Chen *et al.*, 2017).

A practical approach to help faculty to enhance their teaching skills is to develop educator training programs for healthcare educators locally within the institution. Educator training programs provide the opportunity to acquire and practice teaching skills, to receive formal training in educational theory and best practices in educational techniques, to work with a variety of educators, and to develop useful curricular materials ( Tanner and Allen, 2006; Haramati, 2015; Richards, Sinelnikov, and Starck, 2018). The structure of an educator training program can take a variety of forms but should ideally provide a combination of experiences (e.g. shadowing, teaching observation, mentoring) that maximize the benefit to the trainee while also structured in a way that allows them to maintain their existing professional obligations ( Zumwalt and Dominguez, 2019).

While exceedingly useful and practical, educator training programs are rare in medical schools or other healthcare professional schools. One reason for this may be because most medical educators are not experienced or proficient at program design or implementation, so the barrier to creating the programs is significant. This article offers practical, actionable resources for developing and evaluating effective goal-oriented medical educator training programs.

## Program Design

### Articulate Clear Program Goals

The first principle for developing a successful training program is to articulate clear, concrete program goals because these drive the design, implementation, and evaluation of the program. Program goals articulate the reasons the program exists and the mechanisms by which it will make the desired impact. These goals should ideally be articulated as nested hierarchical program objectives: broad *superordinate* goals which encompass focused *intermediate* goals, which in turn drive the action-oriented *subordinate* goals that determine what activities should be built into the program ( Cook, 2010; Höchli, Brügger and Messner, 2018). In addition to driving the program activities, the goals also determine what outcomes should be measured and how to evaluate program effectiveness.

The *superordinate goals* of the program are the overarching objectives of the program and are typically the aims that originally inspired the development of the program. Some examples of superordinate program goals of an educator training program may include: “Improve the skills of medical educators for teaching in an integrated curriculum”; “Improve educators’ skills at providing feedback to learners,” or “Improve educators’ skills at effective interactive teaching.”

*Intermediate goals* articulate how the superordinate program goal will be accomplished by providing a general course of action in the context of the program and its participants ( Höchli, Brügger and Messner, 2018). These should be concrete and focused enough to clearly translate to action, essentially providing a roadmap to accomplishing the superordinate goal. A practical tip for designing appropriate goals is to design goals that are SMART: specific, measurable, achievable, relevant and time-bound ( Bjerke and Renger, 2017). For example, to address the superordinate goal: “Improve the skills of medical educators for teaching in an integrated curriculum,” the following intermediate goals are appropriate: (1) The program will enhance educators’ ability to articulate the application of their basic science topic to clinical practice; and (2) The program will train educators in a variety of techniques to increase interactivity in the classroom.

Finally, the *subordinate goals* of the program precisely describe the activities that trainees will undertake to accomplish the intermediate goals. Some activities implied by each of the intermediate goals listed above are described in Appendix 1. Notice that the list of activities for each intermediate goal is distinctly different, despite the intermediate goals serving the same superordinate goal.

In addition to driving the program activities, clearly articulated program goals ensure that all participants (trainees, mentors, and directors) have the same understanding about the intent of the program and individuals’ roles within the program. This is especially important for complex programs with multiple types of participants. Many training programs bring together individuals from different backgrounds (e.g. different departments or specialties), roles (e.g. students and faculty) or educational backgrounds and perspectives (e.g. PhD scientists, clinical practitioners, and administrators). By having a shared understanding of the program goals, the various individuals will more clearly understand their respective roles within the program.

### Allow the Career Development Objectives of The Trainees to Inform Program Activities

There are a variety of career or skill development goals that may motivate individuals to take the time and effort to voluntarily participate in an educator training program ( Cornelius, Gordon and Ackland, 2011). For example, lecturers may wish to improve their evaluation ratings, educators may wish to become more effective in small group interactive teaching and feedback, and many faculty may wish to create a teaching portfolio for promotion or awards ( Baldwin, Gusic, and Chandran, 2010). For example, useful outcomes for trainees may be documentation of skills such as effective lectures or lesson design, creating a record of teaching experience, or an explicitly crafted statement of teaching philosophy. Program directors should design the structure of the program to be flexible enough to allow individuals to pursue their personal goals. Directors can ascertain the motivations of individual trainees early in the process, incorporate activities that benefit individual trainees’ motivations, and encourage trainees to articulate clear timelines to accomplish concrete outcomes ( Cornelius, Gordon and Ackland, 2011).

### Take Advantage of Complementary Methods for Program Evaluation

It is necessary to put forethought and planning into the program evaluation early in the program design phase to ensure that appropriate observations and data are collected ( Cook, 2010). For full evaluation of a program, data often has to be collected even before the program begins to assess how the program grows and changes or to assess its impact on the participants. Designing clear and concrete program goals facilitates planning useful summative and formative assessments. These assessments then drive the choice of data collection tools that will be used to measure the accomplishment of the goals ( Appendix 2). A practical nuance that should not be overlooked is the need to ensure availability of resources for data collection, such as the tools and the time and personnel resources required to collect and analyze the data.

Program evaluations typically investigate the effectiveness, feasibility, and acceptability of the program as designed and as implemented ( Newcomer, Hatry and Wholey, 2015). These terms are defined below, and concrete examples of what data to collect to address each category of question listed in Appendix 2.

• Program *effectiveness* refers to whether the program is effective at achieving the intermediate and superordinate program goals and what its impact was on the trainees ( Appendix 1) ( Menix, 2007). These types of evaluations especially must be planned well in advance as they often require collection of data even before the program begins. Some questions to consider include: What pre-program data should be collected to enable appropriate pre- vs. post-program comparisons? Is it necessary to perform a needs assessment with the program trainees at the start of the program? What types of data should be collected throughout the program to facilitate appropriate analyses when the program is complete?
• The *feasibility* of the program refers to the ease or difficulty with which the program is implemented, maintained and sustainable ( Bowen *et al.*, 2009; Sanders, 2018). Evaluation of feasibility evaluates the fidelity of implementation, or the degree to which the program is implemented as intended by the developers. This requires examining the availability of the resources required to run the program as well as barriers to its success. For example: How much time is required to run the program, and how many individuals are required to run it? Were there barriers to the success of the program, or did trainees experience barriers to accomplishing their personal goals?
• The *acceptability* of the program refers to the degree to which the program is perceived as useful, relevant, and appropriate for participants and for the institutional environment. This reflects both the level of demand for the program and the likelihood that it will continue to be supported by the institution. These can be measured by examining (1) the adherence and satisfaction to the program by the participants and (2) the perceptions of the relevance and effectiveness of the program by participants in the program and individuals in the organization ( Sekhon, Cartwright and Francis, 2017).

### Don’t Reinvent the Wheel

There are many skills that are essential to the program development and implementation process but may not necessarily be the expertise of individuals who choose to develop and direct programs. Fortunately, many institutions offer core resources that directors can use for program planning, execution, and evaluation. For example, it can be helpful to consult program evaluation experts and statisticians early on to refine the evaluation plan and determine if the institution subscribes to tools for data collection or data analysis. Contacting regulatory offices (e.g. Institutional Review Board) during the design phase can help avoid common programmatic pitfalls. Local experts can also act as advisors, such as librarians to help with literature searches, established educators to advise on educational research methods and theoretical frameworks, and researchers to contribute methodologies unfamiliar to the directors. The program itself may also be able to use existing content (e.g. Massive Open Online Courses, or MOOCs) or curricula available through MedEd Portal ( www.mededportal.org) as part of its programming.

### Collaboration as a means of success

Collaborative relationships can foster the development of strong and healthy programs. For example, programs can be run by a team of collaborating directors with a diversity of experience and expertise. This practice makes it easier to overcome challenges, allows each individual to contribute to the effort in ways in which they are already skilled, and allows the program to benefit from the resources of more extensive personal networks. Working in a team also distributes program responsibilities across multiple busy schedules and creates an accountability system to meet deadlines and milestones in a timely manner.

Collaborating with individuals with diverse perspectives is especially important in a program designed to teach trainees how to be excellent educators. Excellent teaching can manifest in many ways, including robust knowledge of the topic, excellence in communication skills or organization, thoughtfulness in lesson design, and dynamic personality and enthusiasm. Recruiting mentors and participants who exhibit a variety of strengths as educators allows the program to showcase a variety of models of teaching excellence. The ideal group of collaborators consists of both clinicians and basic scientist medical educators, and within those groups the individuals should represent a variety of specialties and types of teaching expertise.

## Program Implementation

It is tempting to believe that once a program has been planned, the work of program design is complete. However, it is likely that the intended plan for the program will not play out as expected, particularly in the pilot year. Once the program begins, unexpected changes in circumstances or observations made through ongoing formative evaluation may modify the program activities or timeline. It is also likely that some aspects of the designed plan will not be feasible, or that unexpected barriers will arise. Two major themes that contribute to the successful implementation of new programs are flexibility of program execution and clear and consistent communication.

### Intentional flexibility

A major recommendation is to intentionally design a program whose goals can be accomplished in a number of ways. This allows the program to adapt to unanticipated scenarios and also, importantly, to allow individuals to participate while continuing to fulfill their primary professional obligations ( McCullough, Marton and Ramnanan, 2015). Some mechanisms to facilitate flexible programming include flexible scheduling practices, program activities that can be accomplished in multiple ways, and reasonable program deadlines. For example, rather than setting a rigid program schedule, ask both trainees and mentor participants to commit to a certain number of hours of work per month, or to complete target activities by certain reasonable deadlines. Another strategy is to minimize in-person meetings by scheduling video conference calls using technology such as Zoom or Skype. When in-person experiences are preferred or required, prioritize flexibility in scheduling. For example, if a trainee participant is expected to shadow a clinician educator to “observe how basic science principles are applied in the clinical setting” ( Appendix 1), provide the opportunity to choose between a number of individuals or from a variety of different educational opportunities (e.g. didactic lessons, inpatient or outpatient clinics, Grand Rounds, etc.). The individuals may then determine for themselves both the pairing and the scheduling that best suits their needs.

### Communication

Regular clear communication is essential to the success of a complex program with numerous participants. Frequent communication (e.g. updates, troubleshooting, and feedback) keeps the program running smoothly and enables all participants to remain clear about the goals, expectations, and deadlines of the program as it progresses. Frequent communication also enables rapid redirection of effort as necessary to ensure that program and individual goals are achieved in a timely manner. Finally, it is essential to maintain regular clear communication between participants (directors, trainees, and mentors) to ensure that all are maximally productive. Directors and mentors should continuously observe the trainees’ progress in the program and provide constructive feedback on how to further enhance their knowledge, competencies and skills. Directors and mentors from different fields (e.g. clinicians, basic scientists, educators, researchers, etc.) can help each other by bridging communication and expertise gaps.

## Program Evaluation and Dissemination

As described above, program objectives determine what evaluations and assessments should be made during the program. Program evaluation can make use of qualitative and quantitative data, each with its own tools and methods for measuring and evaluation. Qualitative data are non-numerical data that describe the qualities or characteristics of a phenomenon, while quantitative data are those data that can be expressed numerically. Mixed methods analyses use both types of data ( Creswell *et al.*, 2011). Collaborating with expert program evaluators from the outset facilitates the evaluation process.

### Qualitative assessment

Qualitative data analysis is both an art and a science and it is beyond the scope of this paper to thoroughly explore its nuances. In general, this type of analysis defines outcomes through exploration and analysis of words and language used by respondents. Common methods for data collection include surveys, questionnaires, written documents (e.g. journals, answers to open ended questions) and verbal exchanges such as interviews that are later transcribed for analysis. Topics that may be explored through qualitative data analyses include the program’s impact on the trainees’ knowledge, skills, and attitudes and the program participants’ satisfaction with program activities. Extensive literature exists elsewhere on topics such as theoretical frameworks, sampling and data collection practices, and data analysis methods used in qualitative data analysis ( Creswell *et al*. 2011; Artino *et al.,* 2014; Moser and Korstjens, 2017; Moser and Korstjens, 2018; Bennett, Barrett, and Helmich, 2019).

It is important to keep in mind some logistical realities of qualitative data analysis. Text-based analyses require the researcher to first organize these data through transcription, cleaning, and labeling of the relevant text (e.g. survey responses, transcriptions of interviews), all of which can be quite time-consuming. There are a number of qualitative analysis computer programs that exist, but these have limitations in that they are able to search text for words but not meaning ( Johnson, Dunlap and Benoit, 2010; Zamawe, 2015). Given the time-consuming nature of this type of data analysis, it is useful to plan ahead for the data management and analysis stage by budgeting either time for the process or finances to hire personnel trained in these skills.

### Quantitative assessment

Quantitative data and the associated analyses are typically more familiar to biomedical researchers than are those of qualitative analyses. Some opportunities for quantitative data collection in program evaluation include counts (e.g. of participants, encounters, etc.) or durations (e.g. of experiences, time to completion, etc.). Another commonly used type of semi-quantitative data is that of numeric rating scales that are built into surveys. In these scales a qualitative characteristic such as an attitude or opinion is quantified by pre-assigning numbers or visual analogies to levels within the scale ( Sullivan and Artino, 2013; Moser and Korstjens, 2018). The most commonly used type of rating scale is the Likert scale, which allows the respondent to express the degree to which they agree or disagree with a sentiment and in which the central value of the scale is neutral [e.g. confidence (extremely to not at all), agreement (agree to disagree), quality (excellent to poor), etc]. Assumptions that are built into this type of ratings scale is that the strength of an attitude is linear along the entirety of the potential range, and that attitudes can be numerically measured both honestly and consistently. Though there is controversy about the statistical validity of applying quantitative analysis methods to this type of data, these methods are valid provided certain statistical criteria are met ( Harpe, 2015).

### Dissemination

At the conclusion of the program it is important to disseminate information about the program and the participants’ experiences with a broader audience so that the program outcomes benefit the field. Two general categories of information that may be disseminated are (1) the impact on the trainees and mentors, and (2) the outcomes of the program development process. In the former category one may address measures of program effectiveness in achieving its goals to improve the trainees’ knowledge and skills as educators, how the program impacted and hopefully improved the trainees’ knowledge and skills as educators, and through reports of program acceptability, such as the perception by the mentors of the trainees’ skills or the utility of the program in the broader educational context. Program development outcomes may be shared through reports on the feasibility and acceptability of the program in the current educational environment. In addition, trainees should be encouraged to publish any educational products (e.g. new resources for teaching) that they developed during the program.

Program evaluation results can be disseminated both broadly and locally. Broad dissemination most obviously includes publication in traditional venues such as peer-reviewed journals or MedEdPortal. Another way to reach a broad, undifferentiated audience is to create a portfolio or website where the products of the education training are showcased to potential participants and education experts. Local dissemination may include presenting seminars or workshops at education and career development meetings to target future trainees and mentors. This is useful to share information about the program to increase institutional buy-in for the program or to recruit future trainees.

## Discussion

Educator training programs for medical educators can have a direct positive impact on the careers of existing faculty ( Dominguez and Zumwalt, 2020). Individuals who make the effort to engage in extra training typically do so to fulfill specific goals that are important to them, such as to acquire new skills to augment their career trajectory. For example, lecturers may wish to improve their evaluation ratings, clinician educators may wish to become more effective at giving feedback, or faculty may wish to expand their experience with a variety of teaching methods. Another positive outcome for all faculty is to expand their network of connections with the educators at their local institution.

The benefits of educator training programs are numerous, but the reality is that faculty who pursue this additional training likely must do so while simultaneously maintaining their current professional responsibilities. Therefore, it is important for program directors to be mindful of these personal motivations and to maximize the opportunities for trainees to accomplish these goals. The principles emphasized in this technical report prioritizing clear and frequent communication, regular assessment, and intentional flexibility in program design allow programs to adapt to the needs and motivations of their participants.

## Conclusion

Development of local educator training programs is a practical solution to the issue in medical education that many faculty lack formal training in teaching. There are many scenarios in which medical educators can benefit from additional pedagogical training, especially in an environment in which medical curricula are rapidly evolving. New curricular models, new instructional design approaches, student feedback, and personal initiative may all motivate an educator to pursue further training.

This article provides practical advice for individuals who want to collaborate to develop educator training programs at their own institution, particularly for individuals not experienced in program design and implementation. The basic themes of successful program development are to plan ahead, be willing to be flexible, allow program design to drive the structure of the program evaluation and find effective collaborators. In the planning phase it is essential to carefully articulate the goals of the program and how to measure accomplishment of these goals. This step drives all subsequent work as it determines the structure of the program, what instruments to use to collect data, and the data analysis and dissemination plan. The development of flexible educator training programs that address the needs of the participants and the institution can positively impact the careers of the trainees, mentors, collaborators, and directors, and ultimately the educational mission of the institution.

## Take Home Messages

• Educator training programs are a practical solution to the challenge in medical education that many faculty members need or desire more formal training in teaching.
• This article provides guidelines for individuals unfamiliar with program design to develop and evaluate an effective educator training program.
• A volunteer faculty development program should be flexible enough to allow participants to fulfill their existing professional obligations while fulfilling their individual educator development goals.
• Best practices for successful educator training programs include planning the program goals and evaluation early in the process, emphasizing flexibility of design, and using collaboration to encourage a diversity of expertise.

## Notes On Contributors

Isabel Dominguez PhD co-directs the Basic Medical Sciences course in the Physician Assistant program at Boston University School of Medicine. She is a basic scientist that directs summer research programs and career development programs for trainees from undergraduate students to postdoctoral fellows. ORCiD: https://orcid.org/0000-0001-9617-4141

Ann C. Zumwalt PhD directs the medical gross anatomy course at Boston University School of Medicine and is a core faculty member in the Vesalius Program, a training program for biomedical science graduate students training to become future medical educators. ORCiD: https://orcid.org/0000-0002-4557-4273

## Appendices

### Appendix 1: Examples of subordinate goals (program activities) implied by named intermediate goals

**Table:**

Intermediate goals	Subordinate goals: Program activities to accomplish the intermediate goal
The program will enhance educators’ ability to engage learners with a variety of experience and expertise	Program trainees will: • Shadow educators who model excellent teaching to audiences unfamiliar to the trainee • Design and present a scientific lesson for audiences with three distinctly different levels of prior knowledge (e.g. lay people, medical students, experienced clinicians). • Receive, analyze, and apply feedback from each audience on their effectiveness in communicating the scientific lessons. • Receive and apply feedback from two different mentors on the design and delivery of one lecture for healthcare students.
The program will train educators in a variety of techniques to increase interactivity in the classroom.	Program trainees will: • Learn and practice three different instructional design methods for eliciting learner responses to questions • Learn and practice the methods of turn and talk and think-pair-share to elicit interactivity in the classroom • Be trained on Audience Response System technology and design a lesson in which they use this technology in a classroom setting

### Appendix 2: Data collection recommendations to address various types of goals

**Table:**

Question	How to analyze	Examples of type of data to collect
Program evaluation: Effectiveness of the program at achieving stated goals		
Superordinate goals: Were the desired outcomes of the superordinate program goals reached? Is the program successful for the target population?	Quantitative or qualitative comparison of intended outcomes as described in the program goals with actual outcomes	Quantitative (e.g. Likert scale) or qualitative analysis of trainees, mentors and directors’ surveys, interviews, focus groups or journals
Superordinate goals: How did the trainees’ perception of education and a career in education change?	Pre- and post-program measures of the trainees’ attitudes about teaching as a component of their career	Before and after the program assess trainees’: • Attitudes about teaching diverse audiences • Attitudes about different types of instructional design (e.g. lecture, flipped classroom, etc.) • Interests in education as a full time/part-time career
Intermediate goals: What did the trainees learn? (skills, knowledge)	Assess the skills that the program is intended to enhance both before and after the program	Assign trainees to create a lesson for a given audience before and after the program. Compare the length, content, and focus of the lesson and the types of teaching tools employed in the lesson
Subordinate goals: What did the trainees learn? (e.g. familiarity with teaching techniques and technology, lesson design, etc.)	Pre- and post-program measures of the trainees’ knowledge of teaching techniques and practices and skills using these approaches.	Before and after the program assess trainees’: • Familiarity with a variety of teaching techniques (e.g. interactive teaching techniques, leading discussions, giving feedback, assessment etc.) • Comfort level and proficiency using common educational technologies (e.g. audience response systems, learning management systems)
Program evaluation: Effectiveness of program of having intended impact on the career development of the trainees		
What is the perceived utility of the program for the trainees’ career goals?	Investigate the perceptions of the trainees about the anticipated impact of the program on their own career. Investigate the perceptions of the mentors about the potential impact of the program on the trainees’ career.	Surveys, focus groups, or interviews after the program to ask what knowledge and skills the trainees gained and how these will be useful to their future careers. What novel opportunities became available as a result of the program? What are the trainee and mentor perceptions of the impact of the program for the trainee’s career?
How productive was the program?	How many publications or other useful resources were produced by the program?	Document how many of the following were produced as a result of the program: • Publications • Curricular innovations • Novel curricular materials • Grants
Program evaluation: Feasibility of the program implementation		
Was the recruitment process adequate to attract appropriate participants?	Compare ideal applicant pool to actual applicant pool Compare ideal mentors to actual mentor pool	Describe the ideal applicant pool and mentors (number of individuals, rank, specialty, teaching experience and skills, etc.) If actual applicants differed from the ideal pool, what about the institutional reality or recruitment process caused this to occur?
How appropriate are the data collection methods for intended purposes?	Compare data collected with data required to answer intended questions	Document the utility of data measurement methods, collection instruments and modalities (i.e. technologies available at the institution) to measure outcomes as intended
How attainable are the program goals?	Honesty document revisions of the intended goals to those that were actually achieved	Document whether the analysis of outcomes performed (goals) was completed as intended. If not, why?
Was the program coherent and logistically practical to implement by the directors?	Honestly document and compare the successes and challenges in implementing the program.	Document: • Time required to recruit trainees and mentors • Financial and time resources required to run the program • Unanticipated institutional barriers to running the program as planned • Resources, training, and expertise needed to manage and implement the program
Were the deadlines for benchmarks met?	Compare intended timelines/deadlines to those accomplished	Document intended and actual timelines for components or phases of the program. If timelines were not accomplished, why not? What was the impact on the program and participants?
Are there barriers to program success?	Document barriers that were beyond the control of the program directors and trainees.	As applicable: • What factors prevented individuals from applying to the program? • What factors slowed the pace of the trainees’ progress in the program? • What factors prevented trainees from completing the program as planned?
What is the likelihood that the program will continue to be in demand? (sustainability)	Investigate the perspectives of individuals who may participate in or support the program in the future.	Document the number of individuals who express interest in participating in the future Document whether relevant administrative offices express support for the program
Program evaluation: Acceptability of the program - Adherence by participants (participation)		
Did trainees engage in activities as intended in the program design?	Compare intended activities to those that occurred	Document the intended and actual descriptions, timing, and duration of the program activities. Document percent participation in the activities.
Did trainees adhere to data collection as intended in the program design?	Compare planned data collection methods and deadlines to those that occurred	Document for all data collection instruments (e.g. surveys, journals, interviews, etc.): % returned Completeness of returned instruments Time spent completing reports
Program evaluation: Acceptability of the program - Perceptions (satisfaction)		
How well were the sessions/activities planned?	Qualitative assessment of the satisfaction with the activities/sessions	After each activity/session, ratings for the following: • Session overall • Facilitator knowledge, organization and delivery • Perceived usefulness/ effectiveness of exercises
Were the trainees satisfied with their experience?	Quantitative or qualitative comparison of satisfaction outcomes as described in the intermediate program goals	Satisfaction surveys of: • Overall outcomes for trainees • Overall satisfaction with the program. • Effectiveness of the modules of the program • Mentors’ and directors’ knowledge and advice • Amount of time spent in the program activities
