# Supplementary material for: Developing a Practical Medical Educator Training Program: Meeting A Need in Faculty Development
Source: MedEdPublish (2016). 2020 Dec 4;9:271. [Version 1] doi: 10.15694/mep.2020.000271.1 (PMC10697581; doi:10.15694/mep.2020.000271.1)
Supplement: Peer review history

# Reviewer report: Reviewer response for version 1

**DOI:** 10.21956/mep.20197.r31255

**Published:** 2021-01-06

**Authors:** Megan Anakin

**Affiliations:** 1 University of Otago

This review has been migrated. The reviewer awarded 3 stars out of 5 Thank you for sharing your experiences and tips about how to develop educator training programmes. I appreciate the practical strategies suggested for programme design, implementation, evaluation, and dissemination. While I appreciate that this article is written for novices in program development, I am wondering if including information about the role and contributions of individuals with educational expertise might enhance this article. The general structure of possible faculty development programmes is well described but examples of possible pedagogical content is missing. For example, given the example goals stated in the second paragraph of the programme design section, I’m wondering if examples of teaching methods and learning activities could be given to illustrate how the programme will benefit educators. Providing examples of how your team has evaluated your programme would also help the reader better understand how to apply the principles at their institution. Another benefit of presenting evaluation data will be to support your claims about the usefulness of the principles that are promoted in this article.To enhance this article, please consider providing references, further details, or specific examples to substantiate the claims made in the sentences with the words ‘many’ and ‘numerous’.I would be very happy to read and review a revised version of this article.

Reviewer Expertise:

NA

No decision status is available

# Reviewer report: Reviewer response for version 1

**DOI:** 10.21956/mep.20197.r31254

**Published:** 2020-12-10

**Authors:** Helena Filipe

**Affiliations:** 1 Hospital of the Armed Forces/PL-EMGFA

This review has been migrated. The reviewer awarded 4 stars out of 5 Thank you for the opportunity to review this extensive, systemic paper on how to design, implement and evaluate faculty development programs with practical nuances for clinical educators simultaneously tasked with their clinical duties.Authors provide a good collection of suggestions organised in three sections: Design, Implementation and Evaluation and Dissemination.I would probably couple with the informative tables of the manuscript a visual representation, diagram perhaps, of the above sections with the main subtopics to facilitate the reader having a quicker overview of the paper framework and useful guidelines, principles and resources suggested.I would like to emphasise essentially three aspects this paper focuses:-that clinical educators benefit from being offered the opportunity to enhance their teaching skills and eventually come to the realisation they can expand their practice on medical education. -that authors bring in and explain a goal categorisation, referred in the business world, with an underpinning theoretical framework spotlighting the goal formulation centred around on what and how people would engage allowing for change in behaviour, in “what people ultimately value and aspire to”. “Goal-setting theory states that challenging, specific, and concrete goals (i.e., subordinate goals) are powerful motivators and boost performance in goal pursuit more than vague or abstract goals (i.e., superordinate goals).” Höchli B, Brügger A and Messner C (2018) How Focusing on Superordinate Goals Motivates Broad, Long-Term Goal Pursuit: A Theoretical Perspective. Front. Psychol. 9:1879. doi: 10.3389/fpsyg.2018.01879 Throughout the paper, Authors strengthen the importance of setting clear expectations by clearly communicating goals, objectives and activities needed to achieve and demonstrate them by the end of the program (outcomes), and how this influence shapes all other steps of faculty development programs. The importance of providing clinical educators with learning opportunities to enhance their skills in developing effective goals and objectives, moved our group’s research, we are happy to share HPriorFilipe, M Paton, J Tipping, S Schneweiss, HG Mack, Microlearning to improve CPD learning objectives Volume17, Issue6, December 2020, Pages 695-699-By referencing the paper with rich and updated literature, Authors effectively complement their paper's purpose on facilitating those more interested in continuing their education on faculty development activities, beyond this paper.I am positive this paper will be useful and motivational for clinical and medical educators and medical education departments leadership.

Reviewer Expertise:

NA

No decision status is available
